# Supplementary material for: CRISPR/nCas9-Based Genome Editing on GM2 Gangliosidoses Fibroblasts via Non-Viral Vectors
Source: Int J Mol Sci. 2022 Sep 14;23(18):10672. doi: 10.3390/ijms231810672 (PMC9505638; doi:10.3390/ijms231810672)
Supplement: Supplementary file 1 [file ijms-23-10672-s001.zip › ijms-1905209-supplementary.pdf]

International Journal of *Molecular Sciences*

Supplementary information

CRISPR/nCas9-Based Genome Editing on GM2 Gangliosidoses Fibroblasts via Non-Viral Vectors

Andrés Felipe Leal [1], Javier Cifuentes [2], Valentina Quezada [2], Eliana Benincore-Flórez [1], Juan Carlos Cruz [2], Luis Humberto Reyes [3], Angela Johana Espejo-Mojica [1], Carlos Javier Alméciga-Díaz [1,*]

[1] Institute for the Study of Inborn Errors of Metabolism, Faculty of Science, Pontificia Universidad Javeriana, Bogotá 110231, Colombia

[2] Department of Biomedical Engineering, Universidad de los Andes, Bogotá 111711, Colombia

[3] Grupo de Diseño de Productos y Procesos (GDPP), Department of Chemical and Food Engineering, Universidad de los Andes, Bogotá 111711, Colombia

* Correspondence: cjalmeciga@javeriana.edu.co; Tel./Fax: +57-1-3208320 (ext. 4140/4099)

Content

Supplementary Table S1. List of primers used in this study……………………………………………………………3
Supplementary Figure S1. MTT and LDH assays…………………………………………………………………………4
Supplementary Figure S2. Nitric oxide determination……………………………………………………………………5
Supplementary Reference...………………………………………………………………………………………………..6

Supplementary Table S1. List of primers used in this study. The sequences for MfeI and MluI enzymes are highlighted in yellow and green, respectively. *Sequences correspond to Leal & Alméciga, 2022 [1].

Purpose	Target	Forward (5′ to 3′)	Reverse (5′ to 3′)
HEXA ORF amplification	*HEXA*	CGATCAATTGATGGCAAGCTCCAGGCTT	ATCGACGCGTGGTCTGTTCAAACTCCTG
HEXB ORF amplification	*HEXB*	CGATCAATTGATGGAGCTGTGCGGGCTG	ATCGACGCGTCATGTTCTCATGGTTACAA-TATCCAGC
*Homologous recombination assay	AAVS1-Out	TTCGATTGGAGTCGCTTTAACTG	-
	CMV	-	AGCTCTGCTTATATAGACCTCC

Supplementary Figure S1. MTT and LDH assays. The figure shows the % of cell viability of GM2 fibroblasts after their interaction with 25μg/mL/0.05mg/mL MNPs@Ag-pD/BUF-II:liposome for 48 hours. ** $p \leq 0.01$.

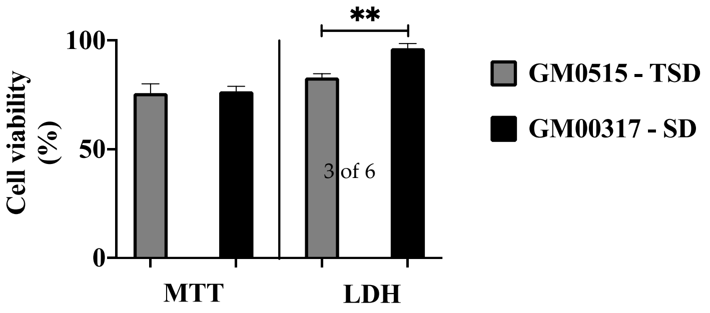

Supplementary Figure S2. Nitric oxide determination. The figure shows the effect of the long-term CRISPR/nCas9-based genome edition on GM2 fibroblasts. Positive control corresponds to the incubation of fibroblasts with 1µM lipopolysac-charide Escherichia coli O111:B4.

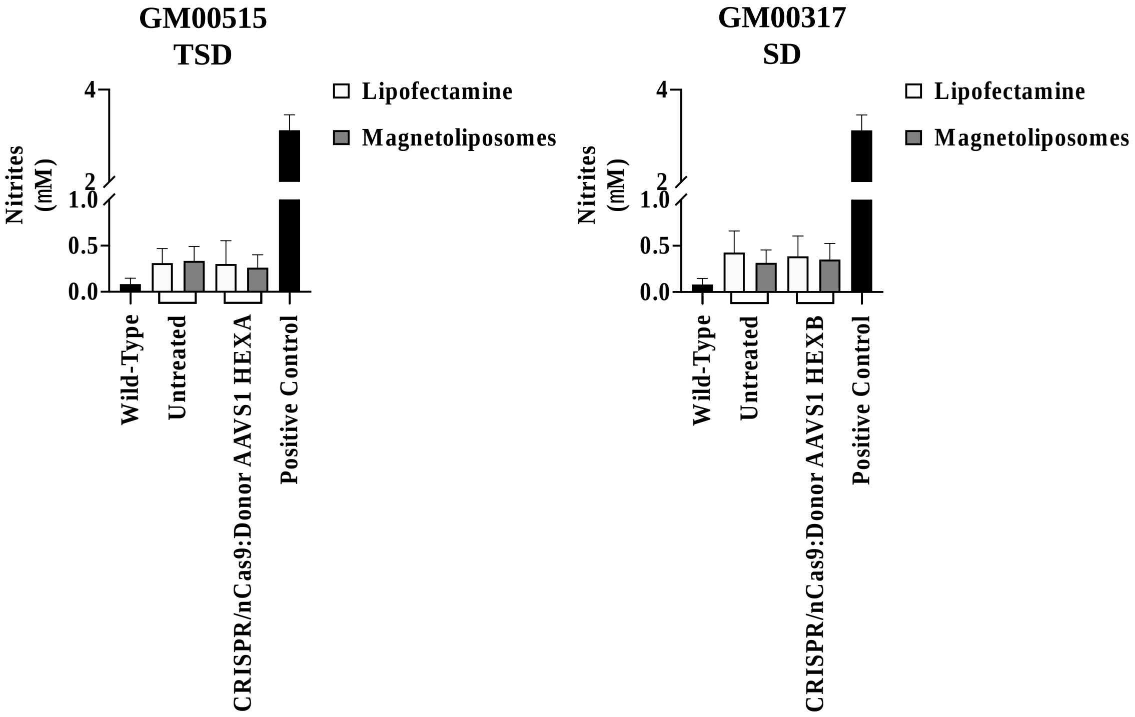

Supplementary Reference

1. Leal, A.F. and C.J. Alméciga-Díaz, *Efficient CRISPR/Cas9 nickase-mediated genome editing in an in vitro model of mucopolysaccharidosis IVA*. Gene Ther, 2022.
